# Optimising PCI by Intracoronary Image-guidance

**DOI:** 10.3389/fcvm.2022.878801

**Published:** 2022-05-13

**Authors:** Mirvat Alasnag, Waqar Ahmed, Rasha Al-Bawardy, Owayed Al Shammeri, Sinjini Biswas, Thomas W. Johnson

**Affiliations:** ^1^Cardiac Center, King Fahd Armed Forces Hospital, Jeddah, Saudi Arabia; ^2^King Faisal Cardiac Center, King Abdulaziz Medical City, Ministry of National Guard Health Affairs, King Saud bin Abdulaziz University for Health Science, Jeddah, Saudi Arabia; ^3^Suleiman Alhabeeb Hospital, Riyadh, Saudi Arabia; ^4^Bristol Heart Institute, Translational Health Science, University of Bristol, Bristol, United Kingdom

**Keywords:** percutaneous coronary intervention, intravascular ultrasound, optical coherence tomography, guidance, optimization

## Abstract

Evidence to support the use of intracoronary imaging (ICI) in guiding percutaneous coronary intervention (PCI) is growing, with observational and randomized controlled trials demonstrating a benefit in acute procedural and clinical outcomes. ICI provides an opportunity to guide PCI, detailing the nature of the coronary disease, potentially influencing lesion preparation and stent selection. Following stent deployment, ICI offers a detailed assessment of lesion coverage, associated vessel trauma and stent expansion. Consensus statements have emphasized the role of ICI and detailed the parameters of stent optimization. However, intracoronary imaging is not adopted widely yet. Significant global differences in the uptake of ICI have been reported, with the vast majority of PCI being angiographically-guided. The three major barriers to the implementation of ICI include, in order of impact, prohibitive cost, prolongation of procedure time and local regulatory issues for use. However, it is our belief that a lack of education and the associated challenges of ICI interpretation provide the greatest barrier to adoption. We hope that this review of the role of ICI in PCI optimization will provide a platform for PCI operators to gain confidence in the utilization of ICI to enhance outcomes for their patients.

## Introduction

Contemporary revascularization provides treatment for an increasingly high-risk patient cohort with progressively more complex coronary disease (1). Percutaneous coronary intervention (PCI) provides the majority of revascularization treatment, however, coronary artery bypass grafting (CABG) remains the preferred strategy in complex disease, due to an excess in repeat revascularization and myocardial infarction associated with PCI (2). Regardless of guideline recommendations, many patients are deemed unsuitable for surgery or decline surgical revascularization, and therefore every effort must be made to optimize PCI outcomes.

Evidence to support the use of intracoronary imaging (ICI) in guiding PCI is growing, with observational and randomized controlled trials demonstrating a benefit in acute procedural and clinical outcomes (3). Differences between intravascular ultrasound (IVUS) and optical coherence tomography (OCT) exist, impacting on the selection of modality for particular patient and lesion subsets (see Tables 1, 2). OCT provides significantly superior resolution compared to IVUS and is particularly suited to detailed plaque assessment and stent visualization, which may be more challenging with IVUS. IVUS however provides a higher level of depth penetration in the vessel and therefore is preferred in large vessels such as the left main coronary artery. A significant limitation of OCT is that it requires clearance of blood from the vessel with contrast injection to allow for image generation by near-infrared light. Consequently, the utility of OCT is limited in patients with renal impairment, aorto-ostial lesions and vessels where there is already injury to the wall, and therefore at greater risk of developing a hydraulic dissection with contrast injection. In these patients, IVUS may be preferred over OCT. However on the whole, studies comparing the role of IVUS and OCT confirm their equivalence and this is acknowledged in the most contemporary guidelines for coronary revascularization (Class 2a, level of evidence B) recommendation for use of ICI to guide left main or complex coronary intervention (2, 4–6).

**Table 1:**
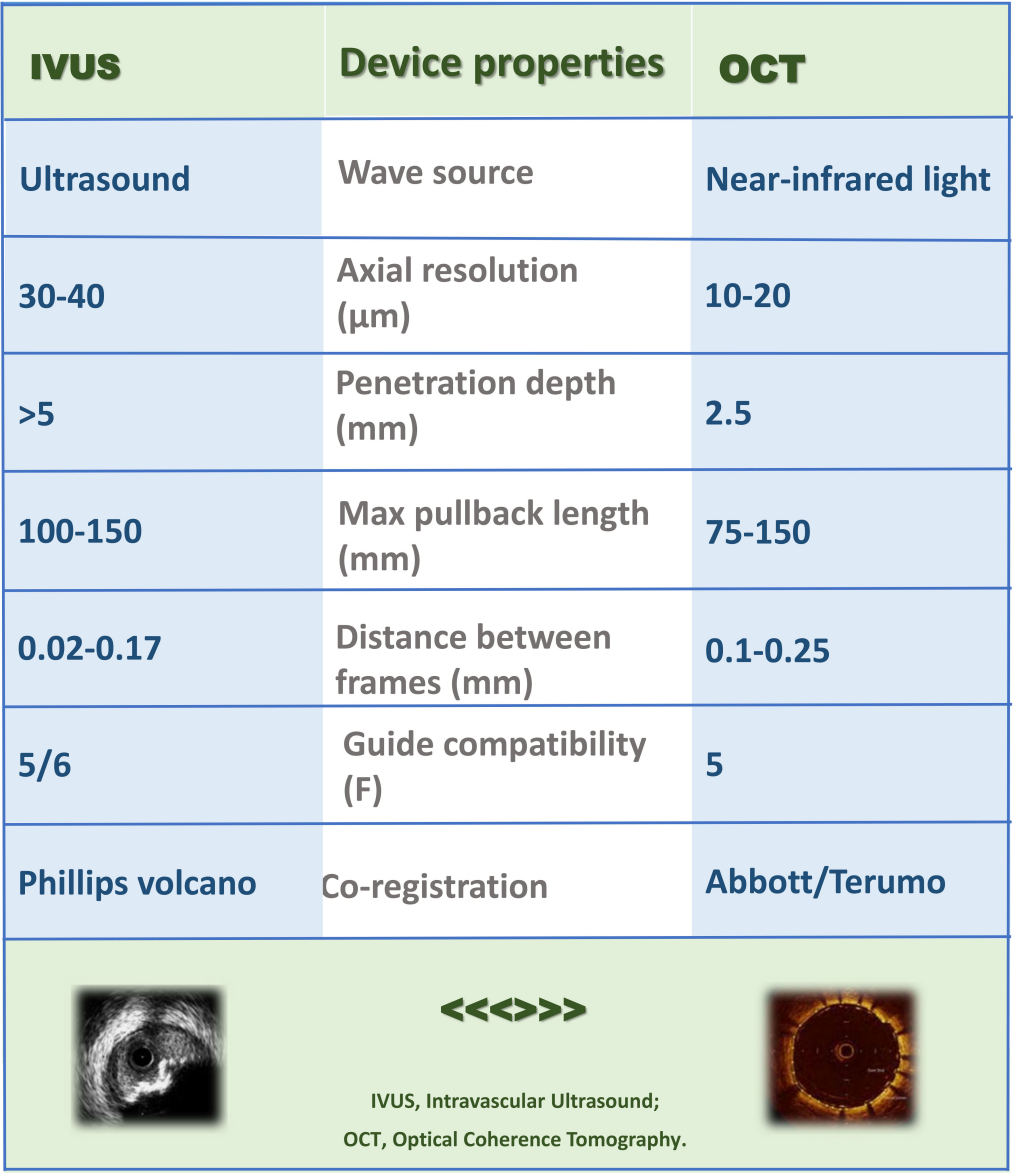
Intra-coronary imaging device characteristics.

**Table 2:**
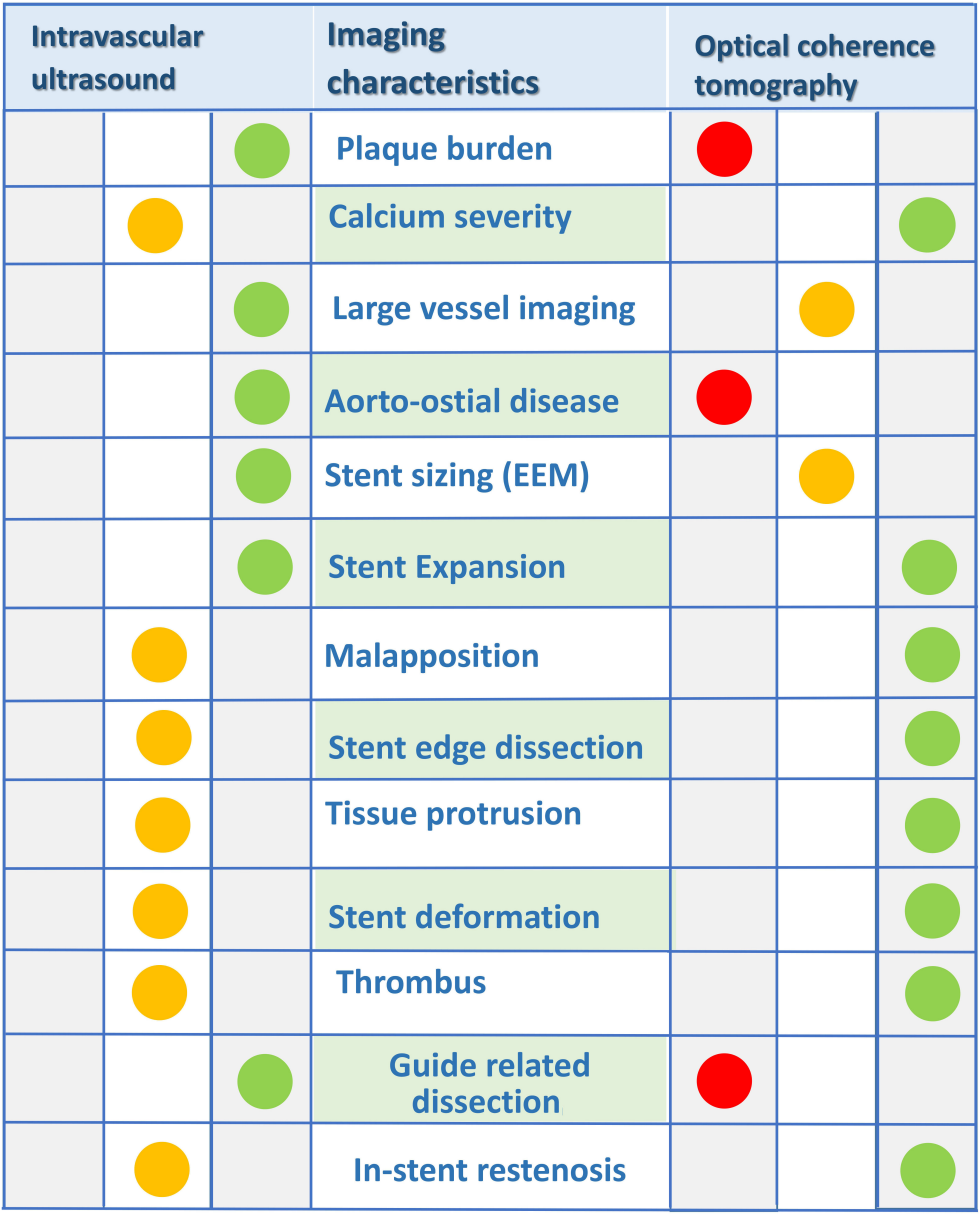
Intra-coronary imaging device characteristics.

ICI provides an opportunity to guide PCI, detailing the nature of the coronary disease, potentially influencing lesion preparation and stent selection. Following stent deployment, ICI offers a detailed assessment of lesion coverage, associated vessel trauma and stent expansion. International consensus on parameters of stent optimization exist but are not adopted widely yet (3). Significant global differences in the uptake of ICI have been reported, with the vast majority of PCI being angiographically-guided (7). The three major barriers to the implementation of ICI include, in order of impact, prohibitive cost, prolongation of procedure time and local regulatory issues for use. However, it is our belief that a lack of education and the associated challenges of ICI interpretation provide the greatest barrier to adoption. We hope that this review of the role of ICI in PCI optimization will provide a platform for PCI operators to gain confidence in the utilization of ICI to enhance outcomes for their patients.

### Utility of Intracoronary Imaging Before Revascularization (Guidance)

Angiography provides a luminal assessment but offers very limited information regarding the composition of the vessel wall. Importantly, it has been shown that quantitative coronary assessment (QCA) derives smaller luminal dimensions than OCT and in turn, OCT provides smaller dimensions than IVUS, but most accurately reflects true vessel size (8).

**Figure 1:**
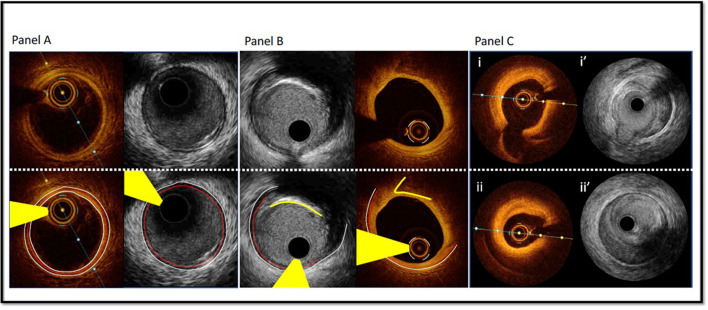
Comparison of Optical Coherence Tomography and Intravascular Ultrasound. Panel A—Matched images of a normal coronary artery using OCT and high-definition IVUS. White dotted line delineates the external elastic membrane, the red dotted line indicates the internal elastic membrane and the white solid line outlines the luminal contour (best identified with OCT). Yellow wedge indicates wire artifact. Panel B—Matched images of fibrocalcific plaque using OCT and high definition IVUS. IVUS facilitates greater visualization of the EEM border due to attenuation of the deep wall structures by OCT (most evident at 3–4 o'clock). OCT's resolution and characteristic sharp edged delineation of calcific plaque (yellow solid line) allows enhanced assessment of overlying fibrous tissue. Panel C—Matched images of a spontaneous coronary artery dissection using high-definition IVUS and OCT. Images i/I' demonstrate intima-medial separation from the EEM at the level of a small sidebranch. Images ii/ii' demonstrate intramural haematoma with some attenuation of deeper structures on OCT and excellent deep wall visualization with IVUS.

ICI accurately defines vessel dimensions and furthermore provides morphological assessment, differentiating normal vessel from various pathologies, including the spectrum of coronary atheroma, vulnerable plaque, thrombus, calcification, vessel ulceration/erosion and dissection. Tissue components are better visualized by OCT given the higher resolution compared with standard gray-scale IVUS (9). This superior resolution makes OCT a better tool to detect dissection, ulceration/erosion and thrombus. However, advancements in IVUS technology with the introduction of higher frequency catheters provides enhanced resolution and maintains the greater depth of vessel preparation compared with OCT (Table 1). Normal vessel architecture consists of intima, media and adventitia (Figure 1). The internal elastic membrane (IEM) is defined as the border between the intima and the media (10). The external elastic membrane (EEM) is defined as the border between the media and the adventitia and is considered the outer vessel boundary for the purpose of vessel measurement (10). Plaque components attenuate both ultrasound and near-infrared light, with calcium blocking IVUS and advanced atheroma obscuring delineation of IEM and EEM by OCT (Figure 1, Panel B).

### Plaque Composition

**Figure 2:**
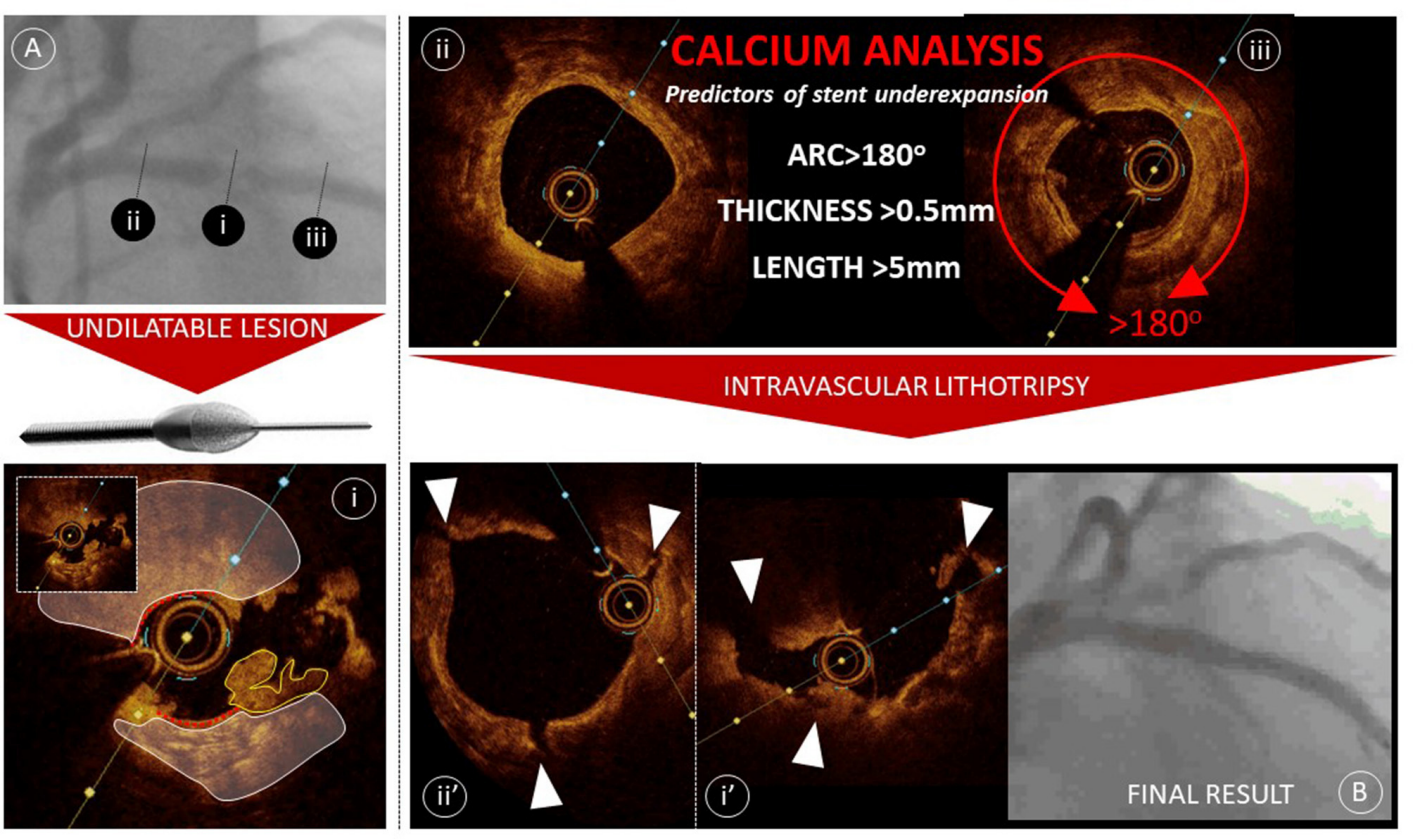
Calcium modification guided by OCT. Panel A—demonstrates a severe mid-left anterior descending artery lesion crossable by an interventional wire but undilatable. OCT imaging is achieved following passage of a 1.5 mm rota-link burr (Boston Scientific) and OCT image i demonstrates heavy burden calcification (white outlined and shaded regions) with evidence of rotablation debulking (red dotted lines) and *in-situ* thrombus (yellow outline). OCT assessment proximal (image ii) and distal (image iii) to the stenosis demonstrate extensive calcification with relative preservation of the lumen area. Intravascular lithotripsy is deployed to ensure adequate calcium modification and repeat OCT confirms effective calcium fracture (white arrows in images ii' & i'. Panel B—An acceptable result is achieved following stent deployment.

Characterization of plaque composition has gained greater attention with the positive clinical outcomes derived using the ULTIMATE IVUS criteria, that encouraged avoidance of stent landing at sites of plaque burden >50% (11). Additionally the arrival of new adjunctive tools to modify calcium have heightened the interventional communities' awareness of identifying significant calcific disease. IVUS and OCT offer differing features of plaque characterization, as described earlier, IVUS provides greater depth of penetration with more consistent visualization of the outer vessel margins, facilitating an assessment of plaque burden. Whereas, OCT provides a more complete appraisal of calcium burden, through the characteristic of near infrared light passing through calcium, providing clear demarcation of calcific disease (Figure 2).

Although severe calcification can be visualized angiographically, it has been demonstrated that OCT and IVUS facilitate significantly greater detection of calcium, with IVUS being most effective. In the same analysis, it was shown that IVUS reverberation artifacts, detected in calcific segments, more commonly associate with a calcium thickness <0.5mm (12). This metric has become important following creation of an OCT-based calcium score that predicts stent under-expansion (Figure 2) (13). It has been shown that in segments of calcific disease with calcium thickness >0.5 mm, with an arc exceeding 180° and extending more than 5 mm, longitudinally, associates with significant stent underexpansion (<70%) in 29.2% of cases. Algorithms are now being devised to combine qualitative and quantitative assessment of calcium to guide the use of adjunctive tools for calcium modification to ensure that vessel preparation is adequate prior to implantation of a stent. An industry-led project implementing the use of OCT to detect calcium and guide treatment resulted in a significant change in practice, with an almost 10-fold increase in the use of aggressive adjunctive tools, including cutting balloon, rotational atherectomy and laser and coronary lithotripsy, however clinical outcome data is awaited (14).

Identification of appropriate stent landing zones requires an awareness of the imaging characteristics of atheromatous plaque disease. As identified earlier, IVUS provides a greater appraisal of the entire vessel wall but the resolution of OCT and the unique features of attenuation and backscatter allows identification of specific tissue components including lipid core arc, lipid core length, fibrous cap integrity and thickness, presence of macrophages, calcifications, thrombus, micro-vessels, and cholesterol crystals (15). Examples of different plaque types identified by IVUS or OCT (10, 15–17) are described below:

- Fibrous plaque is defined as a low-attenuating, signal-rich lesion, with visible IEM and EEM on OCT. A fibrous plaque has medium echoreflectivity on IVUS.
- Lipid-rich plaque (necrotic plaque, fibroatheroma) is defined as high-attenuating, signal-poor lesions covered with fibrous cap on OCT. A lipid-rich plaque has low echoreflectivity on IVUS and are considered attenuated plaques without calcification.
- Fibrocalcific plaque is a combination of fibrous plaque with calcium. It is defined as low-attenuating, sharply delineated, signal-poor lesions. Calcified plaques have high echoreflectivity on IVUS, creating a dark region behind them as ultrasound waves cannot penetrate through calcium, “acoustic shadows”. Calcification can be embedded in the plaque or as a calcified nodule.

Where disease is extensive and a landing zone with plaque burden <50% is not possible, it is important to identify high-burden lipid plaque, as this is most prone to disruption with stent deployment, requiring additional stenting. Where lipid plaque cannot be avoided then it is recommended that stent sizing is undertaken using a luminal dimension and aggressive stent edge postdilatation is avoided.

**Figure 3:**
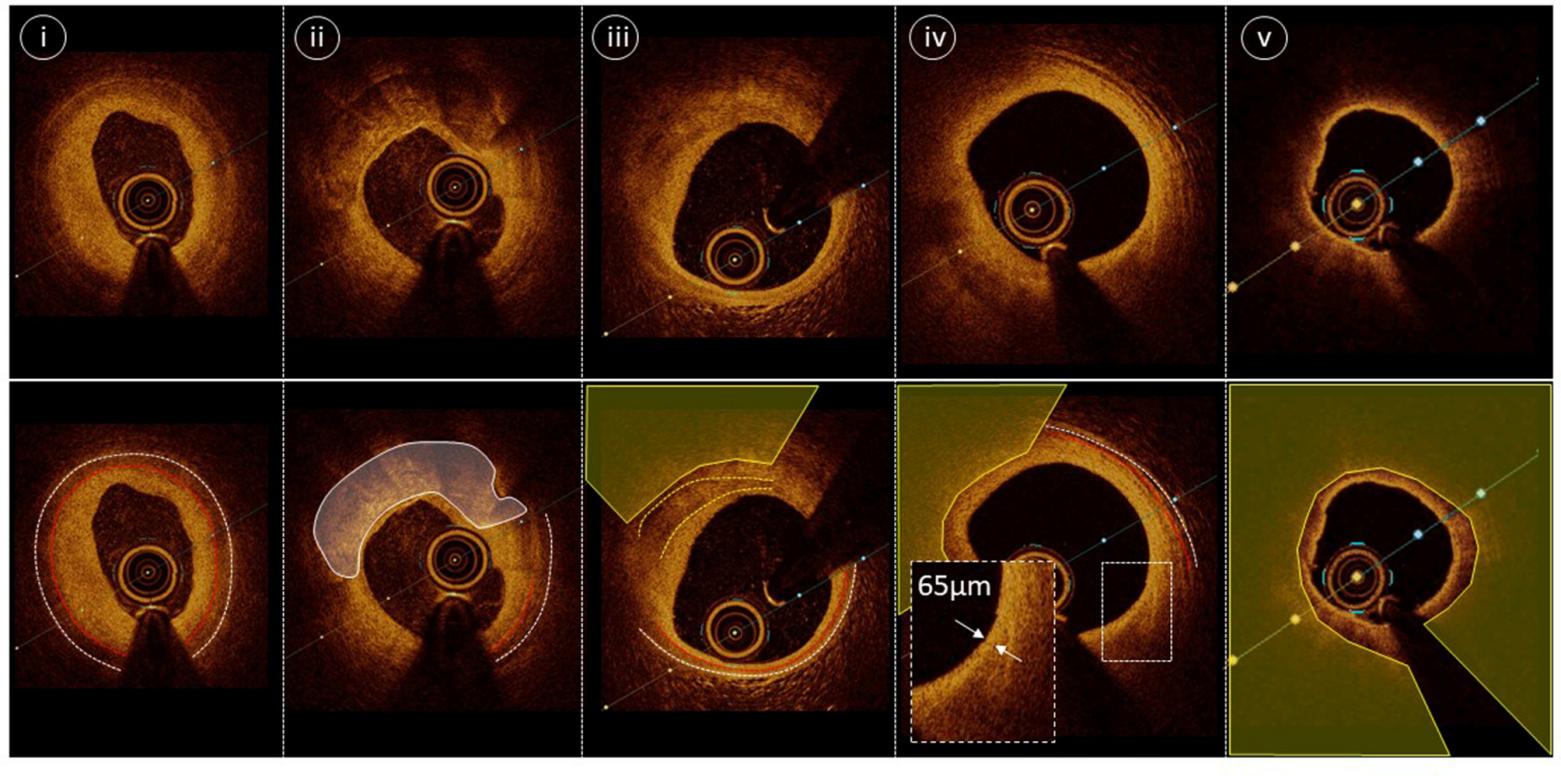
OCT plaque characterization. Image i—represents a fibrous plaque (homogenous bright reflecting signal) with limited attenuation (loss of signal) and well demarcated EEM (white dotted line) and IEM (red dotted line). Image ii—represents fibro-calcific plaque with clearly demarcated boundaries of the calcium (white infilled area). Limited visualization of the EEM & IEM. Image iii—represents layered thick-cap fibroatheroma—layers of fibrous tissue with different reflecting signal highlighted by yellow dotted line and attenuation of signal (yellow infilled area). Image iv—represents a thin-cap fibroatheroma—the expanded area demonstrates a region of fibrous cap thickness <65 μm. Atenuation from the necrotic lipid pool is highlighted by the yellow infilled area. Image v—represents a very high burden lipidic plaque where significant attenuation (yellow infilled area) prevents characterization of deeper vessel wall structures (no EEM/IEM detectable).

Acute coronary syndrome is most likely a consequence of plaque rupture, erosion or calcified nodule resulting in thrombus formation. The management is different based on the finding identified (1–19). Most cases of ST-segment elevation myocardial infarction are secondary to vulnerable plaques that result in plaque rupture or erosion. There are OCT metric criteria for vulnerable plaques, which include: Thin fibrous cap (<65 mm), a large lipid pool, and activated macrophages near the fibrous cap (Figure 3) (15, 19). Of note, intravascular imaging can detect other pathologies in the vessels: including *in-situ* or embolic thrombus, and spontaneous coronary dissection.

### Utility of Intracoronary Imaging During Revascularization (Stent Optimization)

One of the major hindrances to the adoption of intravascular imaging to guide coronary interventions is that there are no universally accepted target criteria for PCI optimization. From the MUSIC criteria in 1998, the TULIP study in 2003, to IVUS-XPL in 2015 and ULTIMATE in 2018, the studies demonstrating improved outcomes have each used different criteria to define stent optimization (11, 20, 21). Table 3 summarizes the various definitions adopted in the different trials over the years (20–23).

**Table 3:**
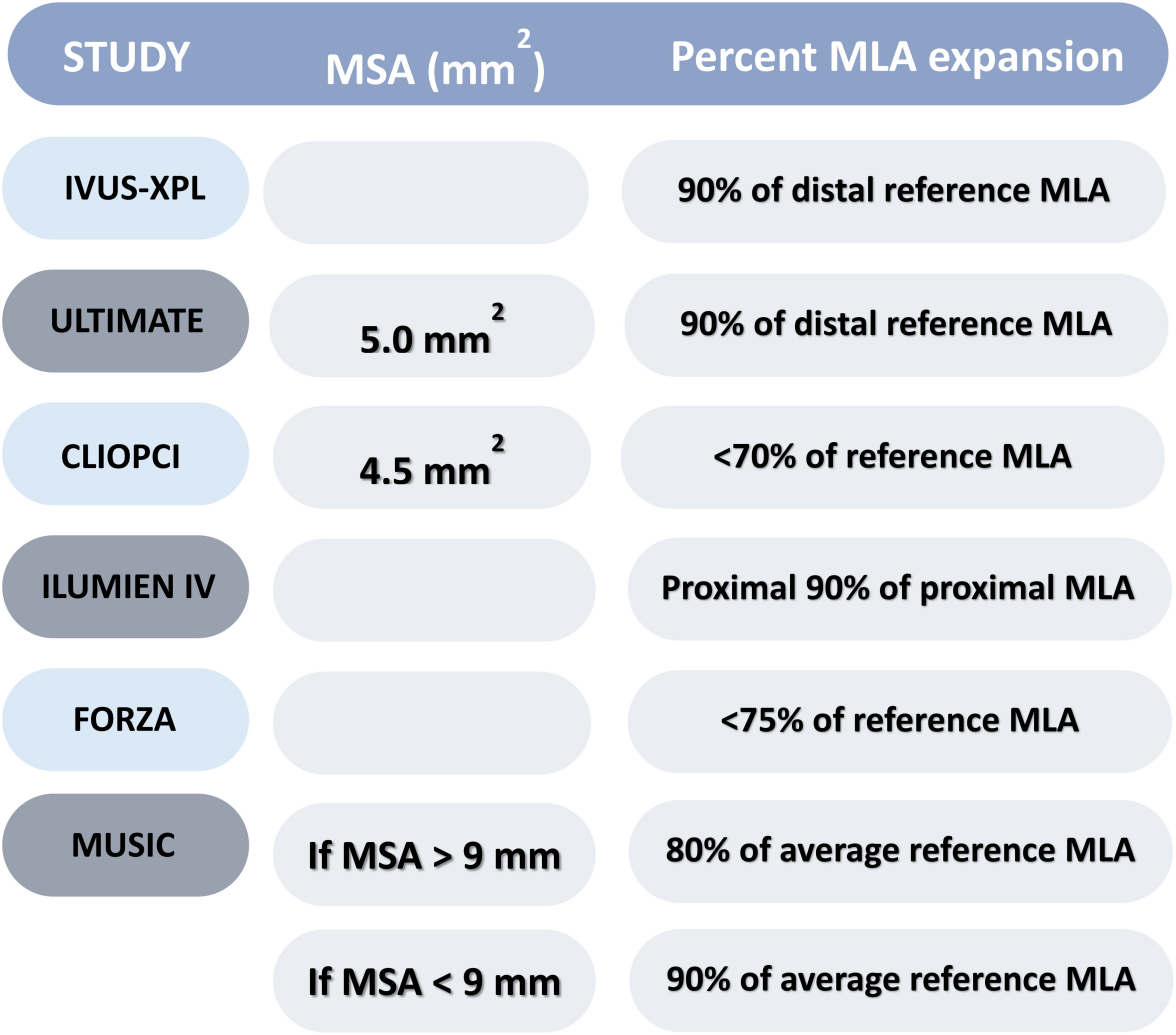
Stent expansion criteria of different studies.

The European Association of Percutaneous Coronary Interventions (EAPCI) Consensus Document on Coronary Imaging recognizes specifically that “there are no uniform criteria regarding recommended targets for PCI optimization in clinical practice” (3). Further confounders are that even in these optimization-dedicated studies, not all patients achieved the ICI-guided expansion targets. For example, only 53% of the ULTIMATE patients met the study criteria for stent expansion.

Stent expansion is based on the total stent lumen area and the minimal stent area (MSA) is the smallest stent area. This usually lies in the distal portion of the stent or in the segment of the stent associated with the tightest segment of a lesion. The etiology of low MSA values are inadequate lesion preparation due to undersized balloons used for pre-dilation, under-appreciation of calcified or fibrotic lesions with inadequate lesion modification, under sizing of the stent and finally inadequate post-dilation of the stent. Commonly, a combination of these factors contribute to suboptimal stent expansion.

**Figure 4:**
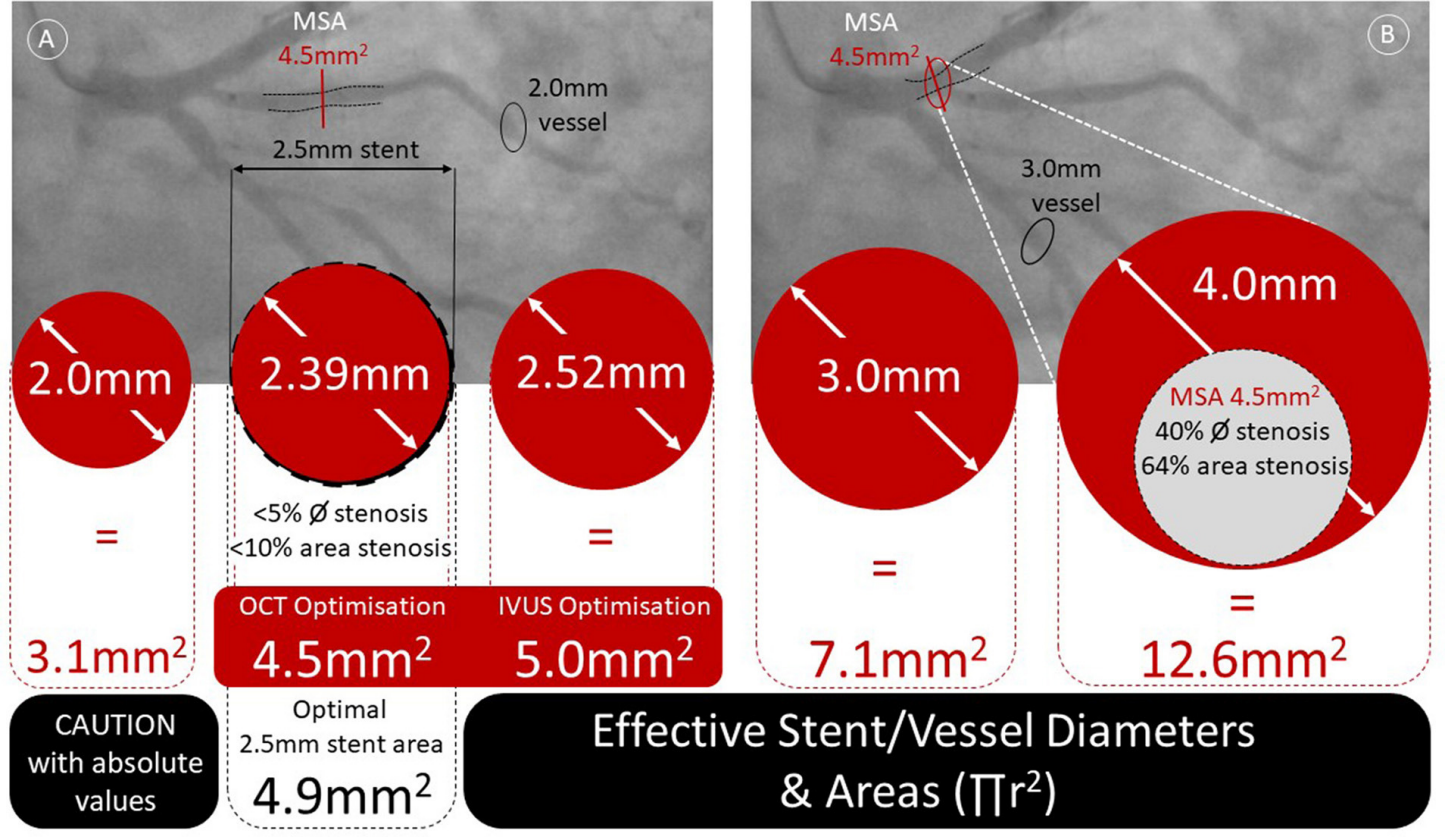
Understanding the relationship between lumen diameter and area with reference to OCT and IVUS optimization criteria. Panel A—demonstrates a 2.5 mm stent (black dotted lines) deployed in a high diagonal branch of the LAD with a minimal residual stenosis angiographically and 2.0 mm distal reference. The 4.5 mm^2^ OCT threshold for optimized stent deployment equates to a 2.39 mm diameter circular vessel (<5% diameter stenosis and <10% persisting area stenosis). Panel B—considers a stent (black dotted lines) deployed in the proximal LAD achieving an OCT estimated minimal stent area of 4.5 mm^2^. The reference vessel diameter of 4 mm equates to a vessel area of 12.6 mm^2^ and therefore the 4.5 mm^2^ threshold results in a persisting diameter stenosis of 40% and area stenosis of 64%).

As evidenced by the existing studies, optimal stent expansion can be assessed by use of an absolute value of MSA or a relative measure commonly derived through comparison of the MSA against either the distal reference area, proximal reference area or an average of both. Consistent with the various optimization criteria, there are numerous cut-offs considered, the EAPCI consensus statement suggested an absolute OCT MSA >4.5 mm^2^, an absolute IVUS MSA >5.5mm^2^ or relative expansion with an MSA/average reference lumen >80% (20). Although use of an absolute MSA value offers clinicians an ease of use, it fails to reflect variation with vessel caliber, where an MSA of 4.5 mm^2^ would equate to >95% stent expansion using a 2.5 mm vessel, but <60% stent expansion in a 4.0 mm vessel (Figure 4). Despite this, the CLIOPCI II registry has recently reported 7.5 year outcome data and confirmed that an absolute 4.5 mm^2^ MSA value continues to predict device oriented clinical events (24). Contrary to this, a recent ADAPT-DES IVUS sub-study undertook a detailed analysis of 10 different stent expansion criteria including the criteria used in the ULTIMATE, ILUMIEN-IV and IVUS-XPL studies. Only the ratio of MSA/vessel area at the MSA site was associated with clinical outcomes (target lesion revascularization or stent thrombosis) at 2 years (25). The cut-off value was > 38.9% but still had only a modest c-statistic of 0.60.

Given that some of the targets in the studies are not easily achievable, the EAPCI Consensus Document on Coronary Imaging recommends a MSA that is 80% of the average (of proximal and distal) reference lumen area (measured by IVUS or OCT) (23). Generally, an MSA of 5.5 mm^2^ is practically used by many interventionalists as acceptable for proximal epicardial vessels and a MSA of 8 mm^2^ for the left main coronary artery. The clinical relevance of acute malapposition remains uncertain. While one OCT based study of stent thrombosis cases suggested that malapposition may be a major predictor of stent thrombosis, this finding has not been replicated widely in either clinical or pathological studies (26). Malapposition remains a relatively common finding, and some studies have shown that more than two-thirds of cases of early malapposition resolved during follow-up (27). Nonetheless, extensive malapposition after stent implantation should be avoided and corrected, when feasible. Acute malapposition of <0.4 mm with longitudinal extension <1 mm or malapposition does not need to be corrected as spontaneous neointimal integration is anticipated. Finally, it is recommended that the proximal or distal stent edges should land at sites with <50% plaque burden.

### Evidence Supporting the use of Intracoronary Imaging

Several randomized trials since the bare metal stent era demonstrated lower restenosis and target lesion revascularization (TLR) rates with IVUS guidance (28, 29). The body of evidence continues to accumulate into drug-eluting stent (DES) era. Adverse events with modern day platforms have become low, making it particularly challenging to demonstrate incremental benefits of intravascular imaging as an adjunct to PCI. As such, many of the randomized trials enrolled complex lesions, such as CTO-IVUS and IVUS-XPL (21, 30). The IVUS-XPL was an investigator initiated randomized trial in Korean patients with long lesions. MACE at 1 year occurred in 5.8% of the angiography-guided PCI and only 2.9% of the IVUS-guided group (*P* = 0.007). Outcomes were a result of a lower risk of ischemia-driven TLR in patients the IVUS-guided group. There was no difference in cardiac death. The CTO-IVUS trial noted no significant difference in the rate of cardiac death between the IVUS-guided group (0%) and the angiography-guided group (1.0%; *P* by log-rank test = 0.16). However, MACE rates were significantly lower in the IVUS-guided group compared with the angiography-guided group [2.6% vs. 7.1%; *P* = 0.035; (HR) 0.35; 95% (CI) 0.13–0.97]. A recent meta-analysis of studies comparing IVUS-vs angiography-guided CTO-PCI demonstrated lower risk of stent thrombosis with reduced stent length and number (31). The IMPROVE trial is an ongoing multinational study evaluating imaging outcomes and TVF at 12 months in those undergoing complex interventions. The study is expected to be completed by 2025 (32). The ULTIMATE study is unique in that it was an all-comer trial with a 1 year follow-up demonstrating significantly lower clinically driven target vessel failure (TVF) in the IVUS group compared with the angiography guided PCI group [4.2% vs. 2.9%; (HR): 0.530; 95% (CI): 0.312–0.901; *p* = 0.019] (11). Several meta-analyses confirmed the role of IVUS in reducing TVR, MACE, cardiovascular mortality and stent thrombosis (33). More recently, Hong et al. elaborated a patient-level analysis from IVUS-XPL and ULTIMATE reporting no difference in 3-year mortality [1% in the IVUS group vs. 2.2 % in the angiography only group (HR 0.43; 95% CI: 0.22–0.84; *P* = 0.011)] (34).

OCT-guided PCI has also been evaluated in multiple studies. The CLI-OPCI registry is one of the largest observational studies that demonstrated a lower rate of cardiac death and MACE with OCT-guided interventions (35). ILUMIEN-I study was a non-randomized trial noting that the initial revascularization strategy was altered when OCT was performed at the start of the PCI in 57% of those enrolled compared with 27% who had imaging after stenting (36). ILUMIEN II and III were randomized trials that demonstrated similar MACE rates with OCT guidance compared with IVUS (4). ILUMIEN III showed that OCT-guided PCI was non-inferior to IVUS in final MSA but did not meet superiority to IVUS or angiography-guided PCI when it comes to MSA. There were small numbers of MACE to detect the differences between OCT, IVUS and angiography-guided PCI. ILUMIEN IV study is ongoing with outcomes anticipated in 2022. The study is assessing the role of OCT in achieving larger post-PCI lumen dimensions and improving clinical cardiovascular outcomes in patients with high-risk clinical characteristics and/or with high-risk angiographic lesions (37). The DOCTORS trial was a randomized trial of 240 patients with non-ST-segment elevation myocardial infarctions (38). In this trial, the post-procedural fractional flow reserve (FFR) was significantly improved with OCT-guidance compared with angiography only. Better stent expansion is the likely reason for this result. The OCTACS study randomized patients with an acute coronary syndrome to OCT-guided revascularization compared to angiography only using newer-generation DES. The OCT arm had fewer uncovered struts at 6 months (4.3 vs. 9.0%, *P* < 0.01) (39). Similarly, the DETECT OCT study revealed better stent coverage at 3 months (7.5 vs. 9.9%, *P*=0.009) with OCT in stable patients (40).

**Figure 5:**
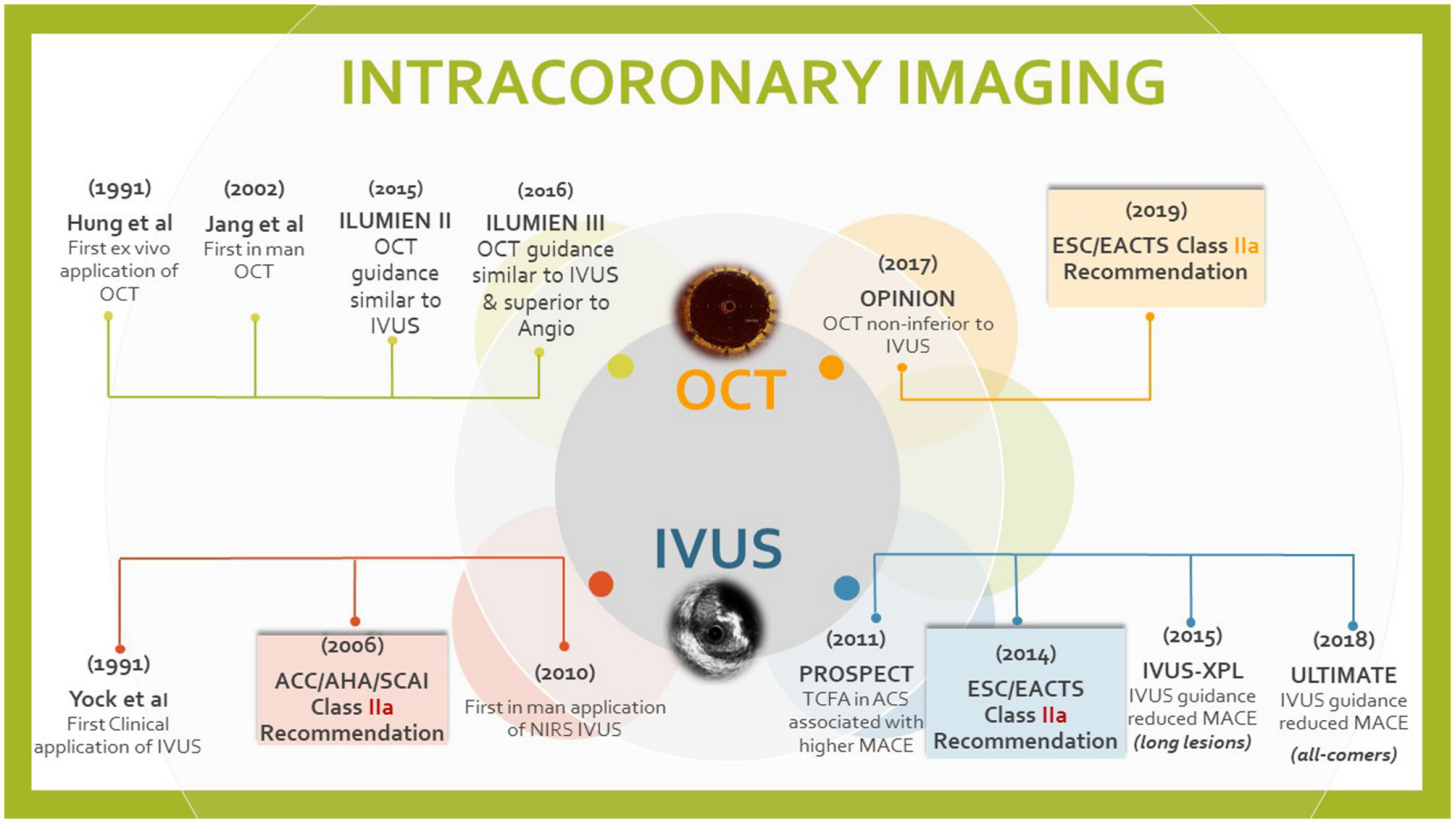
Illustration summarizing the most relevant trials addressing both modalities and the latest guidelines.

Head to head comparisons of OCT and IVUS guided interventions are few. The ILUMIEN-III evaluated the post-stenting MSA following OCT-guidance, IVUS-guidance, and angiography alone in acute coronary syndrome. Minimum and mean stent expansion with OCT was comparable to IVUS-guided PCI. The OCT-guidance group had significantly fewer dissections and malapposition (4). The OPINION trial evaluated results of focal and non-complex disease (lesion length 18 mm) and noted no difference in the TLF at 12 months between OCT or IVUS guidance (5.2 vs. 4.9%) (5). Figure 5 captures the most relevant trials addressing both modalities.

The role of ICI in left main coronary artery (LM) stenting is equally important to address. Observational data has demonstrated a lower MACE (11.3 vs. 16.4%, *P* = 0.04), driven by all-cause mortality at 3 years. The survival benefit may conceivably be a result of the use of larger stents with better expansion and appropriate post-dilatation (41). Similar findings of a 34% mortality reduction were reported from the British Cardiovascular Intervention Society (BCIS) in which 50% of LM PCI procedures were guided by IVUS (42). The MAIN COMPARE revealed a trend toward lower mortality with similar rates of TLR and restenosis when IVUS was used (43). In the EXCEL IVUS sub-study multivariable linear regression denoted only smaller vessel area (*p* < 0.01) and greater calcium angle (*p* < 0.01) were associated with smaller Minimal Stent Area (MSA). Female sex and ostial or body LM lesions correlated with a smaller pre-PCI LM vessel area (both *p* < 0.01) (44). Importantly, an IVUS sub-study of the NOBLE trial demonstrated that in a Western population, the IVUS-MSA criteria of 8/7/6/5 mm^2^ for LM, polygon of confluence (45). The LAD and Circumflex, defined in a Korean population are too small (46). In fact, an MSA ≥13.4.mm^2^ was associated with a 0% TLR rate at 5 years (47). OCT is less frequently considered for LM PCI, particularly for ostial disease, however, the Lemon trial evaluated the feasibility and safety of OCT in LM stenosis. Appropriate stent expansion was seen in 86%, edge dissection in 30% and residual malapposition in 24%. OCT guidance modified the operators' strategy in 26% of the patients. Freedom from MACE was 98.6% at 1 year.

For the average interventional cardiologist in a busy practice, the lack of uniform definitions, expertise in image interpretation and acquisition, reimbursement and the time for image interpretation are the main reasons limiting the wider use of imaging for coronary interventions. Other limitations include the lack of standardization by ethnicity, gender or body surface area. In the future use of artificial intelligence-guided automated measurement of the necessary indices will greatly facilitate the adoption of imaging in routine coronary interventions. Technologies that merge imaging and physiology or both imaging modalities are currently in the pipeline. Ding et al. recently in their *post hoc* analysis reported concordance of post PCI optical flow ratio (OFR) with post-PCI fractional flow reserve (FFR). Simulated residual OFR significantly correlated with post-PCI FFR and stent underexpansion using a single catheter (48). These remain small series and the technology is not widely available. All these tools are yet to be streamlined and integrated into daily practice.

## Conclusion

Intracoronary imaging has a growing role in guiding and optimizing PCI, especially as PCI in more complex and high risk lesion and patient subsets is undertaken. It is our view that imaging should be used prior to, during, and after stent deployment to obtain maximum benefit from the use of an imaging catheter, especially as ICI has important roles to play in all steps of a PCI procedure. With emerging evidence for the benefits of ICI, coupled with increased operator familiarity with ICI imaging modalities, we anticipate volumes of ICI guided and optimized PCI to grow globally in the future.

## Author Contributions

All authors have contributed to the content of this manuscript and reviewed the final version. All authors contributed to the article and approved the submitted version.

## Conflict of Interest

TJ discloses consultancy and speaker fees from Abbott Vascular, Boston Scientific and Terumo. The remaining authors declare that the research was conducted in the absence of any commercial or financial relationships that could be construed as a potential conflict of interest.

## Publisher's Note

All claims expressed in this article are solely those of the authors and do not necessarily represent those of their affiliated organizations, or those of the publisher, the editors and the reviewers. Any product that may be evaluated in this article, or claim that may be made by its manufacturer, is not guaranteed or endorsed by the publisher.
